# Extreme frequency selectivity by synchronized time-modulated metasurfaces

**DOI:** 10.1038/s41598-025-00615-0

**Published:** 2025-05-30

**Authors:** Theodoros T. Koutserimpas, Francesco Monticone, Constantinos Valagiannopoulos

**Affiliations:** 1 School of Electrical and Computer Engineering, Cornell University, Ithaca, NY 14853 USA; 2 School of Electrical and Computer Engineering, National Technical University of Athens, Athens, 15772 Greece

**Keywords:** Materials science, Optics and photonics

## Abstract

Two coupled, time-modulated metasurfaces are governed by Floquet dynamics and exhibit multiharmonic response once they are electromagnetically illuminated. If they get properly synchronized, some of the developed modes may overlap, a feature that changes dramatically the response of the structure with respect to the operating wavelength. This extreme frequency selectivity is theoretically demonstrated for several alternative setups and the reported findings can revolutionize the design of photonic filters, switches or sensors, based on time-varying media.

## Introduction

The interaction of electromagnetic waves with time-varying systems represents a fascinating research topic since the dynamics of the fields can be combined with the dynamics of the media to deliver counter-intuitive properties that cannot be accessed with time-invariant structures. Maxwell equations in unbounded media with permittivity changing with time were first solved in the late 1950s^1^ while the influence of simple boundary conditions on these solutions was identified shortly after^2^. Analytical treatments of space-time wave propagation in materials subjected to temporal variations were proposed several decades ago^3^ and the reflection of beams from an abruptly time-varying medium was also rigorously investigated^4^. The potential of time-modulated texture in adding extra degrees of freedom for wave control has been recently explored further along many different directions, including the study of stability of time-periodic setups^5^, the formulation of temporal parity-time symmetry for extreme energy transformations^6^, and more. Importantly, novel interference mechanisms have been revealed when material dispersion is taken into account^7^ and, more broadly, time-varying metamaterials have opened new opportunities for enriching light-matter interactions beyond the limits of linear time-invariants systems^8,9^.

Time-modulated setups can be used in realizing components with substantial frequency selectivity in their response, which constitutes a long-lasting goal for numerous electromagnetic and photonic devices. In particular, micromechanical nanocantilevers are found to possess unprecedented sensitivity^10^ while nonlinear nanostructures may be employed towards ultrafast all-optical switching^11^ and bio-chemical sensing^12^. Furthermore, optically active tuning of metamaterials exhibiting electromagnetically-induced transparency gives ultrasensitive frequency filters^13^ and guarantee secure communications^14^. Similar aims are served by gratings of coupled rods once anomalous diffraction orders are generated^15,16^ and in bound states in the continuum^17,18^. In addition, efficient frequency-selective metalayers have been proposed to operate as spatial light modulators^19^ while singular waveforms are reported between oscillating walls^20^. Moreover, abrupt waveguide discontinuities exhibit extremely sharp transmission characteristics^21,22^ . Interestingly, sensitive signal detection has become feasible even in quantum layouts, as qubit sensors have been shown to potentially achieve an arbitrary frequency resolution, limited only by the stability of an external synchronization clock^23^.

In this work, we explore the possibility of designing an ultrasharp filter, sensor, or switch based on a time-dependent structure that has been rigorously examined, in a previous study^24^. More specifically, two coupled, time-modulated impedance metasurfaces are excited normally and develop multiharmonic reflecting and transmitting modes at various positive and negative Floquet frequencies. When two of these frequencies (corresponding to different modes) coincide, the overall power of the signal changes abruptly. This modal degeneracy of waves makes the system exhibit extreme frequency sensitivity since the conditions for mode coincidence concur theoretically only at discrete wavelengths. Such a giant selectivity, accompanied by high peak-to-peak amplitude changes, is theoretically demonstrated in several scenarios, while the influence of the admittances and modulation frequencies on the on/off abruptly changing response, is identified. It should be stressed that metasurfaces of temporally oscillating conductivity can be materialized by applying of reversible temperature modulation^25^ or by periodically enforcing mechanical pressure^26^. Therefore, the reported designs are not only extremely efficient but also experimentally feasible and can be incorporated in electromagnetic designs requiring increased sensitivity with respect to the operating frequency.

## Problem formulation

### Multiharmonic reflecting and transmitting modes

**Fig. 1:**
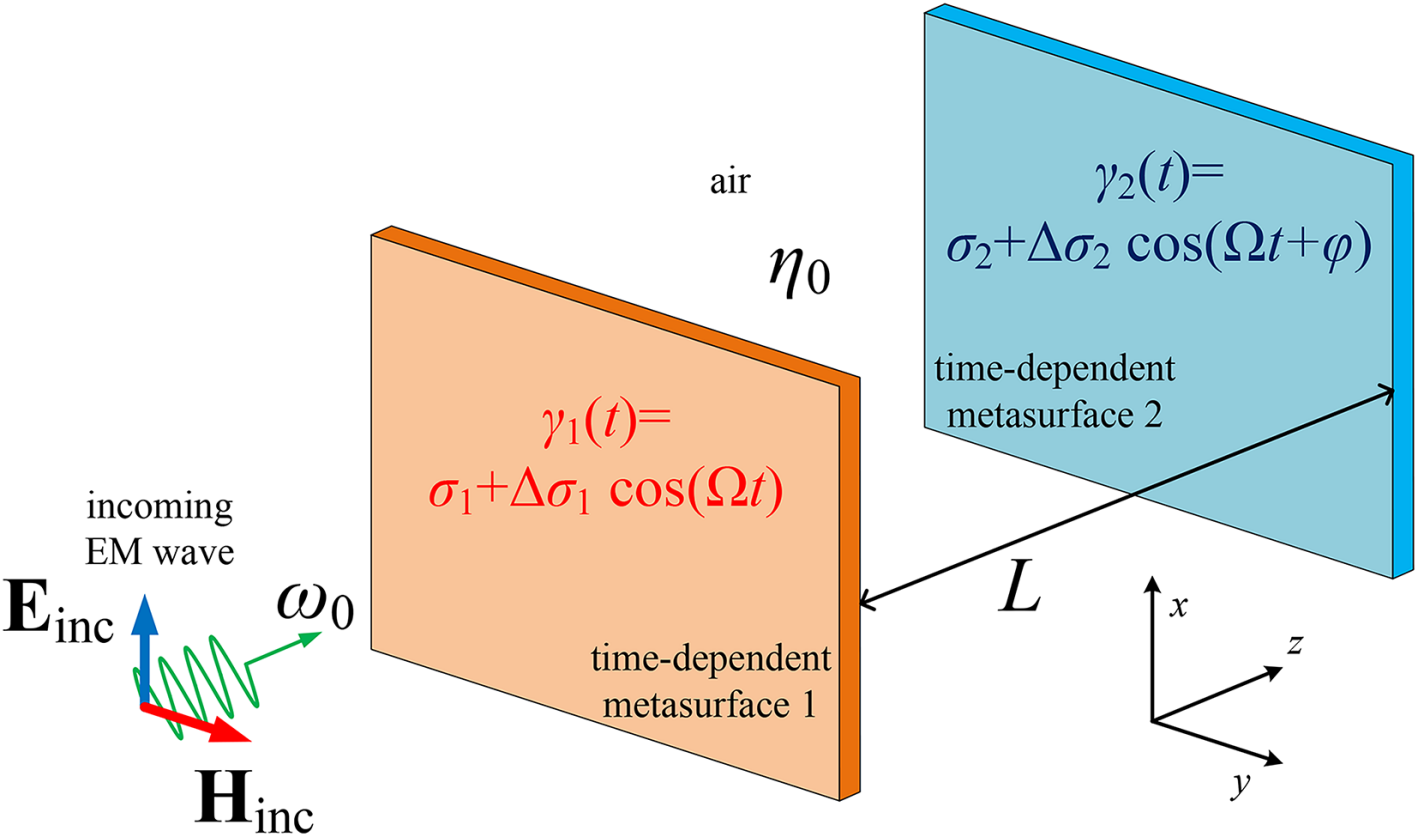
Two coupled metasurfaces are characterized by time-modulated surface admittances $\gamma _1(t),\gamma _2(t)$ and get synchronized to produce signals sharply varying with respect to the primary excitation frequency $\omega _0$. The symbol $\eta _0=\sqrt{\mu _0/\varepsilon _0}$ corresponds to the wave impedance into vacuum. .

Let us consider the setup depicted in Fig. 1. Two metasurfaces at distance *L* apart, are excited by a normally incident electromagnetic wave $\{{\textbf {E}}_\textrm{inc},{\textbf {H}}_\textrm{inc}\}$ of frequency $\omega _0$ traveling along the positive *z* semi-axis, with respect to the local Cartesian coordinate system (*x*, *y*, *z*). The surface conductances, measured in *Siemens*, of the two-dimensional structures are harmonically time-varying with the same modulation frequency $\Omega$. In particular, we denote them as $\gamma _1(t) = \sigma _1+\Delta \sigma _1\cos (\Omega t)$ and $\gamma _2(t) = \sigma _2+\Delta \sigma _2\cos (\Omega t+\varphi )$ for the first and the second flake respectively. The symbols $(\sigma _1,\sigma _2)$ are used for the constant values around which the fluctuations occur and the symbols $(\Delta \sigma _1,\Delta \sigma _2)$ correspond to the respective modulation depths; in addition, the phase difference between the two time modulations is designated as $\varphi$. We assume that $0<\Delta \sigma _1<\sigma _1$ and $0<\Delta \sigma _2<\sigma _2$ so that the conductances are kept positive ($\gamma _1,\gamma _2>0$) for all times *t*; therefore, in the quasi-stationary sense, the setup is passive and lossy. The conductivities are usually expressed in terms of $1/\eta _0$, where $\eta _0=\sqrt{\mu _0/\varepsilon _0}$ is the wave impedance into vacuum.

By following Floquet theory^27^, which is necessary to model the additional dynamics of the time-dependence of the structure, we assume that the produced waves oscillate at the excitation frequency $\omega _0$ shifted by integer multiples of the modulation frequency. More specifically, the reflected ($z<0$) and transmitted ($z>L$) electric fields are found by the Floquet ansatz:

1a $$\begin{aligned} {\textbf {E}}_\textrm{ref}= & \hat{{\textbf {x}}}\sum _{n=-\infty }^{+\infty } R_n e^{+\textrm{i}\frac{\omega _0+n\Omega }{c}z}e^{\textrm{i}(\omega _0+n\Omega )t}, \end{aligned}$$

1b $$\begin{aligned} {\textbf {E}}_\textrm{tran}= & \hat{{\textbf {x}}}\sum _{n=-\infty }^{+\infty } T_n e^{-\textrm{i}\frac{\omega _0+n\Omega }{c}z}e^{\textrm{i}(\omega _0+n\Omega )t}, \end{aligned}$$

where $c=1/\sqrt{\varepsilon _0\mu _0}$ is the speed of light in vacuum. With reference to (1), we denote the frequency of each mode as $\omega _n \equiv \omega _0+n\Omega$. The unknown complex coefficients $\{R_n,T_n\}$ of each mode for $n\in \mathbb {Z}$ are found by imposing the necessary boundary conditions at $z=0,L$; all the details regarding the derivation of the respective expressions are contained in the prequel paper^24^. They call for continuity of the electric fields ${\textbf {E}}$ and discontinuity of the magnetic fields, evaluated via Faraday’s law $\frac{\partial {\textbf {H}}}{\partial t}=-\frac{1}{\mu _0}\nabla \times {\textbf {E}}$, by equivalent surface electric currents proportional to: (1) the local electric fields ${\textbf {E}}$ and (2) the time modulated surface conductivities $\gamma (t)$.

### Mode degeneracy at synchronized regimes

Any time-dependent structure requires extra energy to change dynamically; as a result, the temporal modulation can pump power to the system and may render the whole configuration active. The time-averaged power density carried by the reflected and transmitted waves, which is permitted to be larger than the energy of the incoming excitation wave, makes a meaningful metric to be represented. Indeed, it accounts for the response of the coupled structure across all excited frequencies and from both sides of the formed cavity; in other words, it says how strongly interacts with the background electromagnetic fields. Due to the linear independence between waves of different frequencies $\omega _n \equiv \omega _0+n\Omega$ for $n\in \mathbb {Z}$, the power of the two developed waves can be also expressed as:

2 $$\begin{aligned} P=\sum _{n}\left( |R_n|^2+|T_n|^2\right) , \end{aligned}$$

while the incident electric field is taken with unitary magnitude: 1 *Volt*/*meter*. The harmonics appear at positive frequencies for $n>-\omega _0/\Omega \equiv -r$ and at negative frequencies for $n<-r$; all the assumptions behind the eduction of the formula (2) and the related expressions can be found in our previous study^24^. Moreover, the expression (2) originates from Poynting theorem meaning that sums like those in (1) (electric fields) are multiplied by quantities (magnetic fields) proportional to their complex conjugates. Due to the aforementioned mode orthogonality, only terms of the same frequency survive the time averaging, since they yield time-independent cross products. However, the same happens when the frequency of one mode (index *n*) equals to the opposite of another mode (index *m*); particular care should be taken in these special cases, where the harmonics overlap. In such a scenario, (2) may give an incorrect result since the amplitude of the wave product at $\omega =\omega _n=-\omega _m$ will be modified. The condition of frequency overlapping is easily found:

3 $$\begin{aligned} \omega _n=-\omega _m\Rightarrow \frac{\Omega }{\omega _0}=-\frac{2}{n+m} \Leftrightarrow n+m=-2r. \end{aligned}$$

Note that, in order to satisfy (3), the signs of the two frequencies $(\omega _n,\omega _m)$ should be opposite and, usually, the same happens for the indexes (*n*, *m*). In such a case of mode coincidence, the combined power of the reflected and transmitted signals is given by the modified formula^28^:

4 $$\begin{aligned} P'= \sum _{n,m}\left\{ \begin{array}{c} \left( |R_n|^2+|T_n|^2\right) (1-\delta _{n+m+2r})\delta _{n-m}+\left( |R_n+R^*_m|^2+|T_n+T^*_m|^2\right) \delta _{n+m+2r} \end{array}\right\} , \end{aligned}$$

where the summations are performed for all $n\in \mathbb {Z}$ and $m\in \mathbb {Z}$ unlike in (2) where only summation with respect to $n\in \mathbb {Z}$ occurs. The notation $\delta _x$ is used for Kronecker’s delta (one for $x=0$ and zero for $x\ne 0$). The sum in (4) comprises of two parts, the first of which is identical to that of (2) and gets activated only if the condition (3) is not valid ($\delta _{n+m+2r}=0$) and $n=m$ ($\delta _{n-m}=1$). The second term is nonzero only for those pairs of modes with indexes (*n*, *m*) that satisfy the frequency coincidence constraint (3), namely, $n+m=-2r$. In such a scenario, the reflection and transmission coefficients are added up since they refer to a single operational frequency $\omega _n=-\omega _m$. The complex conjugate appeared in the quantities $(R_m^*, T_m^*)$ is dictated by the spectral form of Parseval’s theorem^29^.

It is remarkable that (4) is valid also at the frequencies $\omega _0=-\frac{n+m}{2}\Omega$, while in their spectral vicinity the response of the system is correctly expressed by (2) and one obtains $P'=P$. Thus, if the ratio $P'/P$ deviates substantially from unity, an extremely selective frequency filter is obtained, with large potential for sensing or switching applications. Therefore, a major objective in the following is to find parametric combinations to realize synchronized metasurfaces with a large difference between the response $P'$ exactly at $\Omega /\omega _0=-\frac{2}{n+m}$ (on) and $P \ne P^{\prime}$ in the neighborhood of this value (off).

According to the analysis above, there are infinite potential frequencies at which such a synchronization between the coupled planar oscillators may occur. However, in the subsequent numerical results, we will focus mainly on the case with $\Omega /\omega _0=2$ in order for the indexes (*n*, *m*) to assign their minimal values while satisfying the condition (3). In other words, if one integer $n\in \mathbb {Z}$ is added to another integer $m\in \mathbb {Z}$, the pairs (*n*, *m*) with minimum $(|n|+|m|)$ that yield a negative sum $(n+m)$ are $(n,m)=(0,-1)$ and $(n,m)=(-1,0)$, both corresponding to the aforementioned frequency. The reason why we require small magnitude for the indexes (*n*, *m*) is related to the series in ((1a), (1b)); these infinite sums are convergent and usually the higher-order terms are weaker than the lower-order ones. Therefore, the synchronized metasurfaces are more likely to exhibit on/off ratio $P'/P$ substantially different from one when the two fundamental modes coincide at $\Omega /\omega _0=2$.

## Numerical results

### Response change at synchronization

**Fig. 2:**
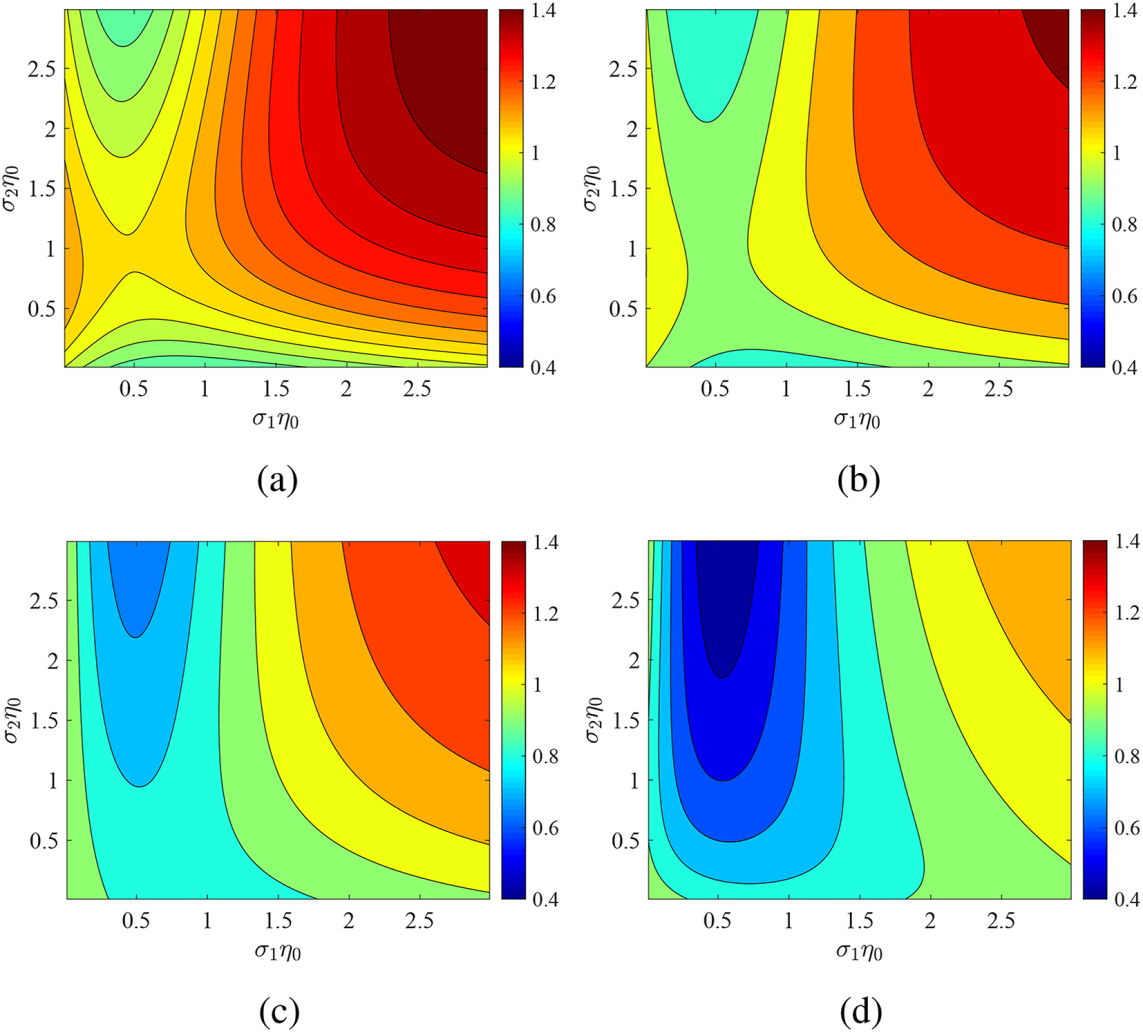
Influence of phase $\varphi$ on the power ratio $P'/P$ at $\Omega /\omega _0=2$ across the average admittances map ($\sigma _1,\sigma _2$) for fixed optical thickness $\omega _0L/c=\pi /2$. (**a**) $\varphi =0$, (**b**) $\varphi =\pi /4$, (**c**) $\varphi =\pi /2$, (**d**) $\varphi =3\pi /4$. Both metasurfaces modulated: $\Delta \sigma _1/\sigma _1=\Delta \sigma _2/\sigma _2=0.9$.

To demonstrate the change in the produced power by the coupled system, we evaluate the ratio of the power $P'$ when synchronization occurs over the respective quantity *P* infinitesimally away from such a regime. A ratio $P'/P$ significantly different from unity means that the sensing and filtering capacity of the structure is large, given the fact that the change occurs at discrete frequencies. In Fig. 2, we represent the metric $P'/P$ on the plane of fixed conductivities $(\sigma _1\eta _0,\sigma _2\eta _0)$ for various phase differences $\varphi$ at $\Omega =2\omega _0$. We notice that in all the four considered cases, significant deviation from unity is observed indicating high amplitude switch. More specifically, for $\varphi <\pi /2$ (Fig. 2a,b) we note that $P'>P$, especially if both conductances are increasing simultaneously. On the other hand, sizable minima of $P'/P$ are detected for $\varphi >\pi /2$ (Fig. 2c,d) particularly if the second metasurface is characterized by high losses.

**Fig. 3:**
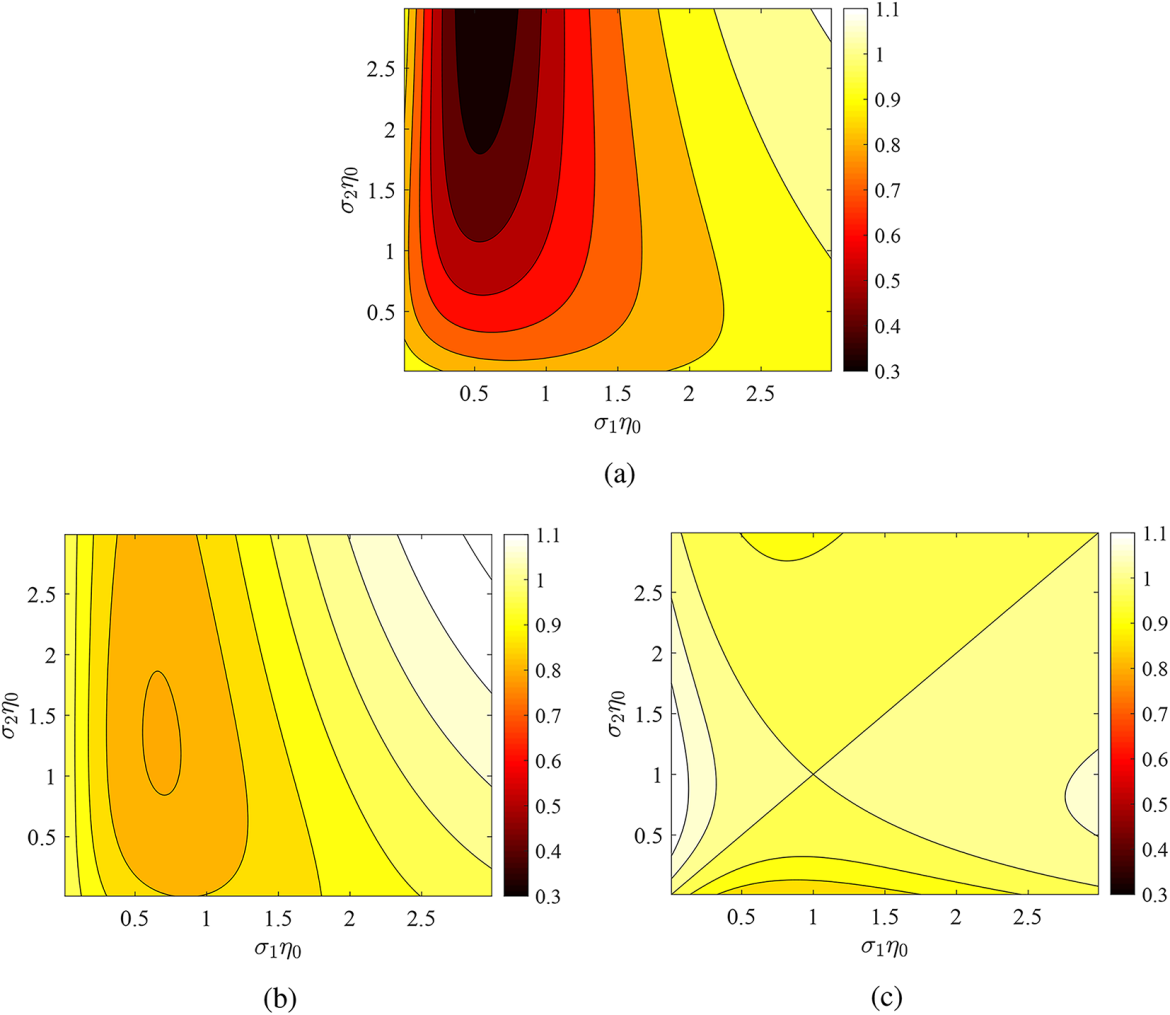
Influence of optical thickness $\omega _0L/c$ on the power ratio $P'/P$ at $\Omega /\omega _0=2$ across the average admittances map ($\sigma _1,\sigma _2$) for fixed phase $\varphi =\pi$. (**a**) $\omega _0L/c=\pi /2$, (**b**) $\omega _0L/c=3\pi /4$, (**c**) $\omega _0L/c=\pi$. Both metasurfaces modulated: $\Delta \sigma _1/\sigma _1=\Delta \sigma _2/\sigma _2=0.9$.

In Fig. 3, we repeat the calculations of Fig. 2 by keeping the phase constant at $\varphi =\pi$ while changing the electrical thickness $\omega _0 L/c$ of the cavity. Both metasurfaces are modulated with the same depth and the most interesting results are observed for the smaller thickness *L* (Fig. 3a) where $P'/P$ is found to take values as small as 0.3, meaning that power is suppressed more than three times once the two sheets are synchronized, compared to operating at an infinitesimally close frequency. For thicker cavities (Fig. 3b), we obtain a shallower minimum of $P'/P$ across the same region as in Fig. 3a while, if one increases *L* further (Fig. 3c), the contrast is lower and mostly $P'>P$.

**Fig. 4:**
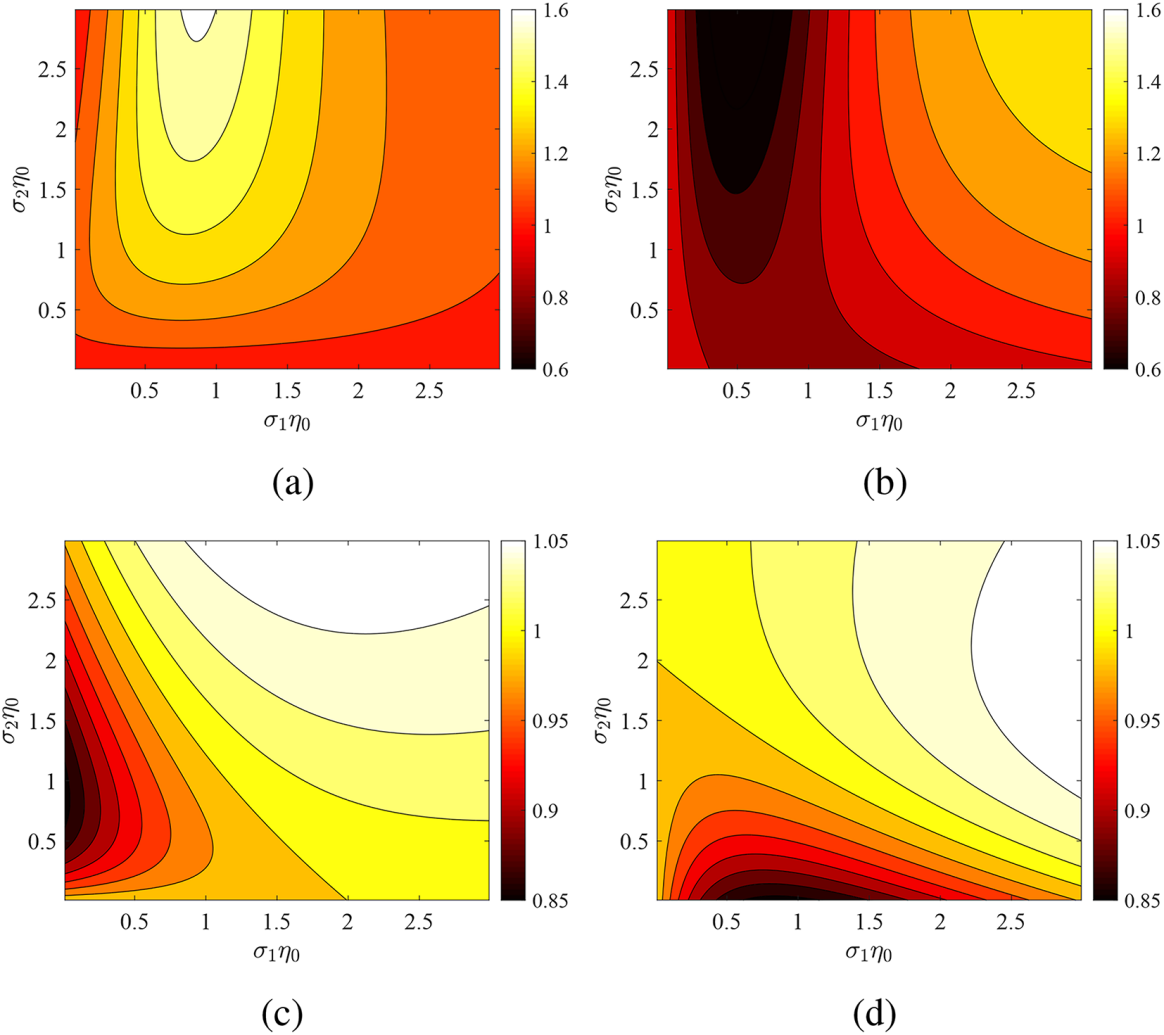
The same as Fig. 3 but for fixed phase $\varphi =0$. (**a**) Only second metasurface modulated with $\Delta \sigma _2/\sigma _2=0.9$, $\omega _0L/c=\pi /2$, (**b**) only first metasurface modulated with $\Delta \sigma _1/\sigma _1=0.9$, $\omega _0L/c=\pi /2$, (**c**) only second metasurface modulated with $\Delta \sigma _2/\sigma _2=0.9$, $\omega _0L/c=\pi$, (**d**) only first metasurface modulated with $\Delta \sigma _1/\sigma _1=0.9$, $\omega _0L/c=\pi$.

In Fig. 4, we show again the ratio $P'/P$ across the map $(\sigma _1\eta _0,\sigma _2\eta _0)$ with $\varphi =0$ and when only one of the two metasurface is being modulated. In Fig. 4a the first metasurface is time-invariant and in Fig. 4b, the second one is kept fixed with conductance $\sigma _2$. In the former case, the metric $P'/P$ is maximized and in the latter it is minimized; however, both scenarios yield a similar on/off power contrast. In Fig. 4c,d, we redo the computations of Fig. 4a,b but for sheets placed at double the distance apart. The ratio $P'/P$ possesses values closer to unity, an indication that the excited waves for $0<z<L$ are destructively interfering each other. Interestingly, the two sets of results seem almost identical to each other with the values of $(\sigma _1,\sigma _2)$ being exchanged. The reported conclusions make another indication that the phase difference $\omega _0L/c$ introduced by the cavity size can act as a controller to the effect of multiharmonic resonances at coupled time-modulated metasurfaces.

### Extreme frequency selectivity

**Fig. 5:**
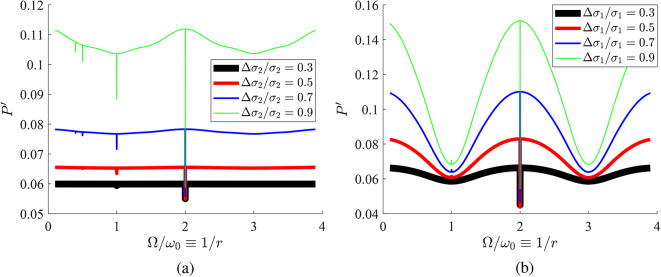
Demonstration of extreme frequency selectivity of the structure for $\varphi =\pi$ and $\omega _0 L/c=\pi /2$. Response $P'$ is represented as function of the frequency ratio $\Omega /\omega _0$ for various modulation depths of the time-dependent metasurface ($\sigma _1\eta _0=0.58$, $\sigma _2\eta _0=5$). (**a**) Only second metasurface modulated, (**b**) only first metasurface modulated.

To gain more insight into the behavior of this time-modulated system, it is also interesting to study its power response as a function of frequency, which is expected to show the effect of modal degeneracy described above. In Fig. 5, we show $P'$ as function of $\Omega /\omega _0\equiv 1/r$ when one of the two flakes changes its admittance with time according to various modulation depths (for $\varphi =\pi$). These results clearly show the extreme frequency selectivity of the structure, originating from the fact that condition for mode coincidence (3) is obeyed only at discrete frequencies. In both Fig. 5a,b, the jumps in the response $P'$ are more substantial for higher normalized modulation amplitudes while the discontinuous output is recorded only for $r>1/2\Rightarrow \Omega <2\omega _0$, consistent with (3). As mentioned above, the synchronization effect gets weaker as $\Omega /\omega _0$ becomes smaller and it gets maximized at $\Omega =2\omega _0\Leftrightarrow r=1/2$. Once the second metasurface changes with time (Fig. 5a), the on/off switching is more powerful compared to the case in which the first metasurface is modulated (Fig. 5b); however, this feature is attributed to the fact that $\sigma _2>\sigma _1$ in this specific example. Finally, as expected, the jumps get more significant for increasing modulation depths.

**Fig. 6:**
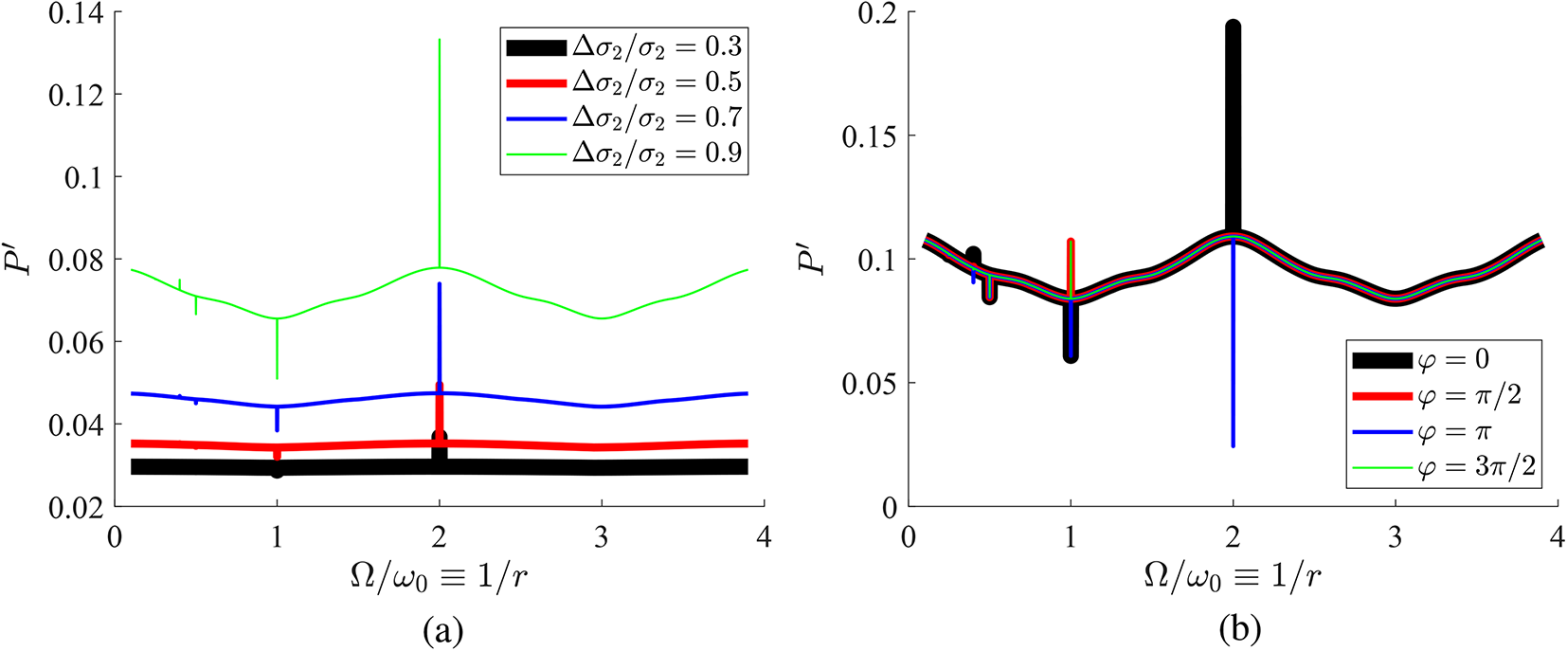
Same setup as in Fig. 5 ($\sigma _1\eta _0=0.58$, $\sigma _2\eta _0=5$) and only second metasurface modulated for: (**a**) various modulation depths and zero phase (**b**) various phases and 0.99 modulation depth.

In Fig. 6a, we represent the frequency response of $P'$ by keeping fixed $\varphi =0$ (different from choice $\varphi =\pi$ for Fig. 5). Once again, the discontinuities emerge distinctly at the frequencies where the two sheets are synchronized but now (unlike in Fig. 5) not only sharp drops but also peaks are recorded. In Fig. 6b, we keep assuming a time-invariant first metasurface and utilize a very high modulation depth for the second one ($\Delta \sigma _2/\sigma _2=0.99$), while considering multiple phase differences $\varphi$. One observes that all the curves, outside of the frequencies where modes coincide, are almost identical and what really changes with $\varphi$ is the direction of abrupt switching (peak or drop). Notably, for specific values of $\varphi$, the discontinuity disappears, a finding that illustrates the control that the phase difference $\varphi$ has over the operation of the proposed setup.

### Experimental feasibility

In general, time modulation of electromagnetic properties is achieved using an external agent. Causality dictates that any temporal change in the refractive index or electric susceptibility directly affects the temporal profile of the material’s losses-and consequently, its conductivity^34^. Consequently, any modulation method employed to induce a temporal change in the refractive index may be used. Several alternative approaches involve mechanical mirroring for water waves driven by the Faraday instability^35^, time refraction of particles spin leading to fluctuating magnetic fields^36,^ as well as acousto-optic light scattering to produce nonreciprocal single-sideband modulation^37^.

In addition, optomechanically induced non-reciprocal transparency and amplification are observed and a significant non-reciprocal phase shift has been also demonstrated^38^ while time-dependent photonic interband transition has become feasible via electrically driven nonreciprocity on a silicon chip^39^. An ideal experimental platform might involve reversible temperature modulation to achieve large contrasts in electrical and thermal conductivities using first-order phase transitions in percolated composite materials^25^. Finally, another approach concerns the application of controlled mechanical pressure, namely, a technique first conceptualized by Edison for the speaking telegraph^26^.

## Discussion

Paired homogenized metasurfaces characterized by time-modulated conductivities while oscillated at the same frequency, are positioned at a certain distance apart and illuminated normally by a plane wave of another frequency. The reflected and transmitted fields are written as weighted sums of waves at various Floquet frequencies; however, if certain harmonics of two different modes coincide, the expression for the overall device response will change. In this sense, abrupt switches in the output of the coupled setup are observed as the synchronizations occur at discrete wavelengths. Such a substantial frequency selectivity is illustrated in numerous configurations that can be employed for filtering and sensing purposes. In particular, high-fidelity filtering has been reported in space-time modulated metasurfaces based on simultaneous frequency translation and angular deflection^30^. Moreover, significant optical nonreciprocity is achievable with help from the extremely narrowband frequency responses materialized via sharp synchronized filters^31^. On the other hand, electromagnetic tagging and cooperative target recognition has been demonstrated with time-modulated grating^32^ while high-quality arrival sensing and detection have become feasible with similar setups^33^.

An interesting expansion of the present work would be to consider different modulation frequencies for each metasurface so that the engineering of synchronization in terms of the coinciding wavelengths, becomes possible. In addition, the peak-to-peak contrast is a quantity that can be maximized by playing with unequal modulation paces and determines the quality of the device as a sensor. Finally, an important next step is to formulate a feasibility map of this sensing performance, across the time-dependent metasurface conductivities. In this way, one can determine optimal on/off operations that correspond to realistic designs, possibly leading to the practical implementation of synchronized photonic components with extreme frequency selectivity.

## Data Availability

The datasets used and/or analysed during the current study available from the corresponding author on reasonable request.
